# Urban rural differences in diet, physical activity and obesity in India: are we witnessing the great Indian equalisation? Results from a cross-sectional STEPS survey

**DOI:** 10.1186/s12889-016-3489-8

**Published:** 2016-08-18

**Authors:** Jaya Prasad Tripathy, J. S. Thakur, Gursimer Jeet, Sohan Chawla, Sanjay Jain, Rajender Prasad

**Affiliations:** 1 International Union Against Tuberculosis and Lung Disease, The Union South East Asia Office, New Delhi, India; 2 Department of Community Medicine, School of Public Health, Post Graduate Institute of Medical Education and Research, Chandigarh, India; 3 Department of Internal Medicine, Post Graduate Institute of Medical Education and Research, Chandigarh, India; 4 Department of Biochemistry, Post Graduate Institute of Medical Education and Research, Chandigarh, India

**Keywords:** Diet, Physical activity, Obesity, Urbanization, STEPS survey, India

## Abstract

**Background:**

The rising morbidity and mortality due to non-communicable diseases can be partly attributed to the urbanized lifestyle leading to unhealthy dietary practices and increasing physical levels of inactivity. The demographic and nutrition transition in India has also contributed to the emerging epidemic of non-communicable diseases in this country. In this context, there is limited information in India on dietary patterns, levels of physical activity and obesity. The aim of the present study was thus to assess the urban rural differences in dietary habits, physical activity and obesity in India.

**Methods:**

A household survey was done in the state of Punjab, India in a multistage stratified sample of 5127 individuals using the WHO STEPS questionnaire.

**Results:**

No rural urban difference was found in dietary practices and prevalence of overweight and obesity except the fact that a significantly higher proportion of respondents belonging to rural area (15.6 %) always/often add salt before/when eating as compared to urban area (9.1 %). Overall 95.8 % (94.6–97.0) of participants took less than 5 servings of fruits and/or vegetables on average per day. No significant urban rural difference was noted in both sexes in all three domains of physical activity such as work, transport and recreation. However, rural females (19.1 %) were found to be engaged in vigorous activity more than the urban females (6.3 %). Males reported high levels of physical activity in both the settings. Absence of recreational activity was reported by more than 95 % of the subjects. Higher prevalence of obesity (asian cut offs used) was seen among urban females (34.3 %) as compared to their rural counterparts (23.2 %). Abdominal obesity was found to be significantly higher among females in both the settings compared to males (*p* < 0.001).

**Conclusions:**

Poor dietary practices and physical inactivity seems to fuel the non-communicable disease epidemic in India. Non communicable disease control strategy need to address these issues with a gender equity lens. Rapid urbanization of rural India might be responsible for the absence of a significant urban rural difference.

## Background

Out of 56 million deaths in 2012 globally, 38 million were due to NCDs, principally diabetes, cardiovascular diseases, cancer and chronic respiratory diseases [1]. These four major NCDs were responsible for 82 % of NCD deaths. Nearly three quarters of these NCD deaths (28 million) occurred in low- and middle-income countries. NCD deaths have increased the most in the WHO South-East Asia Region, from 6.7 million in 2000 to 8.5 million in 2012 [1]. While the annual number of deaths due to infectious disease is projected to decline, the total annual number of NCD deaths is projected to increase to 52 million by 2030 [2, 3].

Unhealthy dietary practices, sedentary lifestyle and obesity have emerged as major risk factors of NCDs [4]. All these risk factors are lifestyle related and are influenced by change from rural to urban lifestyle. Even in rural areas with modernization and advent of mass media, there is gradual shift to urbanized lifestyle. It is important to understand the changing dietary pattern and levels of physical activity in the context of economic and demographic transition that India is undergoing due to rapid globalization and urbanization. India is also witnessing the phase of degenerative disease in the nutrition transition. It is associated with a shift in dietary patterns to more ‘Western’ diets rich in saturated fat, refined foods and sugar and low in fibre [5]. While there are numerous studies from western countries on diet and levels of physical activity in their respective populations, few studies from India have looked at these important risk factors in this changing scenario.

The present study was done as part of a NCD Risk Factor Survey conducted in 2014-15 by Post Graduate Institute of Medical Education and Research, Chandigarh in collaboration with National Health Mission, Punjab under the aegis of Ministry of Health and Family Welfare, Government of India. Thus, the objective of this study was to document urban rural differences in dietary practices, levels of physical activity and obesity in a North Indian state of Punjab.

## Methods

### Study setting and design

Punjab is a prosperous state in northern part of India bordering Pakistan with a population of 2.7 million according to 2011 national census. It ranks second in terms of Human Development Index among all states [6]. A state wide NCD risk factor survey based on WHO-STEPS approach was undertaken in Punjab in 2014–2015. The survey adopted a multistage stratified, geographically clustered sampling approach using the 2011 census sampling frame. In urban areas, a three-stage procedure was followed. In the first stage, wards were selected by Probability Proportional to Size (PPS) sampling. In the second stage, one Census Enumeration Block (CEB) was randomly selected from each sampled ward. In the final stage households were selected within each CEB using systematic random sampling. The rural sample were selected in two stages: the selection of Primary Sampling Units (PSUs), which are villages by PPS at the first stage, followed by the selection of households within each PSU at second stage using systematic random sampling. Out of a total of 100 PSUs, 60 were from rural areas and 40 were CEBs from the urban locality. From each selected PSU, 54 households were selected. The ultimate sampling units were the households and one individual in the age group of 18–69 years residing in the selected household was selected using the KISH method.

### Data collection instrument

A culturally adapted, hindi translated and pre-tested version of the WHO STEP Surveillance (STEPS) questionnaire (version 3.1) was used with minor adaptations [7]. As part of the household survey, socio demographic and behavioural information on tobacco and alcohol use, diet, physical activity, history of chronic diseases, family history of chronic conditions, health screening, and health care costs were collected in Step 1. Physical measurements such as height, weight, blood pressure and waist circumference were collected in Step 2. Biochemical tests were conducted to measure fasting blood glucose, total cholesterol, triglycerides, HDL and LDL in Step 3. In the present article results from Step 1 and Step 2 of the survey are presented.

### Data collection

Data were collected by trained investigators. Height was measured using a SECA adult portable stadiometer. The participants were asked to remove shoes, socks, slippers and any head gear. It was measured in centimetres up to 0.1 cm. SECA digital weighing scales were used to measure weight of the individuals. These were regularly calibrated against standard weight. The participants were asked to remove footwear and socks and weight was recorded in kilograms up to 0.1 kg. Waist circumference was measured at the level of the midpoint between the inferior margin of the last rib and the iliac crest in the mid-axillary plane. The measurement was taken at the end of a normal expiration with the arms relaxed at the sides. A SECA constant tension tape was used to take the measurement to the nearest 0.1 cm.

One serving of vegetable was considered to be one cup of raw green leafy vegetables or 1/2 cup of other vegetables (cooked or chopped raw). One serving of fruit was considered to be one medium size piece of apple, banana or orange, 1/2 cup of chopped, canned fruit or 1/2 cup of fruit juice.

Physical activity was assessed using the Global Physical Activity Questionnaire (GPAQ), which has been developed by the World Health Organization [7]. This questionnaire assesses physical activity behaviour in three different domains: work, transport and during leisure time in a normal active week for the participants. The GPAQ attempts to estimate the total weekly volume of moderate- and vigorous intensity activities (MVPA) in a typical week in these three domains. The duration and frequency of physical activity (min/day) participation in three domains (activity at work, travel to and from places, and recreational activities) over a typical week are recorded. Activities are classified into three intensity levels: vigorous, moderate and light [5]. ‘Vigorous-intensity activities’ are activities that require hard physical effort and cause large increases in breathing or heart rate, ‘moderate-intensity activities’ are activities that require moderate physical effort and cause small increases in breathing or heart rate. A summary estimate of total MVPA in min/day was calculated by combining the activity score of both moderate- and vigorous-intensity activity for each work and recreational activity domain. Participants are further classified into three activity intensity categories (low-, moderate-, or high-intensity activity level) according to their total physical activity per week (MET-minute per week) based on the GPAQ guidelines [8]. Participants are also classified as sufficiently active who exceed the minimum duration of physical activity per week recommended by WHO i.e. 150 min of moderate intensity physical activity or 75 min of vigorous intensity physical activity or an equivalent combination of moderate- and vigorous-intensity physical activity achieving at least 600 MET-minutes per week with each activity performed in bouts of at least 10-min duration [8]. Body Mass Index was calculated using the formula, weight (in kilograms)/ (height in meters squared). Overweight was defined as BMI > = 25 kg/m^2^ and <30 kg/m^2^. Obesity was defined as BMI > = 30 kg/m^2^. Asian cut off for obesity (> = 27.5 kg/m^2^) and overweight (> = 23 kg/m^2^ and <27.5 kg/m^2^) was also used [9]. Cut-offs used for waist circumference were > =90 cm for men and > =80 for women [10, 11].‘ Show cards (pictorial, adapted to the local context) were used to explain to the participants the type of physical activity, servings of fruits and vegetables and salty food intake.

The Scheduled Castes (SCs) and Scheduled Tribes (STs) are groups of historically disadvantaged people in India recognised by the Constitution of India. They comprise about 16.6 and 8.6 % respectively of India’s population (according to the 2011 census). Other Backward Class (OBC) is a collective term used by the Constitution of India to classify castes which are socially and educationally disadvantaged. They comprise of around 41 % of the population. They are considered to be at a less disadvantageous position than SCs and STs. General category is a term used in India to denote a group other than OBCs, SCs and STs and are considered socially, educationally, and economically advanced. Thus they do not qualify for any of the reservation or social welfare benefit schemes.

### Sample size

Sample size of 4609 was calculated using the estimated prevalence of physical activity as 50 %, 0.05 margin of error and 95 % confidence interval. Assuming a response rate of 85 %, sample size was raised to 5400.

### Data analysis

Variables were summarized using mean/median and proportions with 95 % confidence intervals. Chi-square test was used for comparison of proportions across groups and ANOVA test for comparison of means across groups. Kruskal-Wallis test for comparison of medians across groups wherever applicable using Epi Info version 3.5.2. The estimates derived were weighted for age, sex and non-response. Odds ratios (95 % confidence intervals) were calculated to estimate the association of various socio-demographic characteristics with obesity and lack of physical activity.

## Results

Out of 5400, a total of 5127 individuals gave consent for STEP 1 and 2 (response rate of 95 %).

### General characteristics of the respondents

Of 5127 respondents, 2746 (54 %) were women and 2381 (46 %) were men. 65 % of the study sample belonged to 18–44 years age group. Around 60 % belonged to the rural areas which is similar to the urban rural distribution in the state population as per Census 2011. Mean household income of the study population was INR 58833 (approximately 905 USD). Nearly 23 % of the study participants had no formal schooling with only 10 % educated above high school. Distribution by social group shows that 47 % belonged to General category and 38 % to the Scheduled Caste category. About 49 % of the participants were unpaid home makers followed by self-employed (1290, 25 %)-mostly agriculture and non-government employees (957, 19 %) Table 1.

**Table 1:** Socio-demographic profile of study participants in STEPS Survey, Punjab, India

Socio-demographic characteristics		Rural	Urban	Total
		*N* = 3096	*N* = 2031	*N* = 5127
Age	18–44	2046 (66)	1298 (64)	3344 (65)
	45–69	1050 (34)	733 (36)	1783 (35)
Sex	Male	1438 (46)	943 (46)	2381 (46)
	Female	1658 (54)	1088 (54)	2746 (54)
Educational status	No formal schooling	848 (27)	355 (17)	1203 (23)
	Upto primary school	776 (26)	490 (24)	1266 (25)
	Upto high school	1264 (41)	862 (43)	2130 (42)
	Higher education	196 (6)	322 (16)	518 (10)
Social Group	SC	1236 (40)	691 (34)	1927 (38)
	OBC	401 (13)	298 (15)	699 (14)
	General	1409 (46)	1001 (49)	2410 (47)
	Unknown	50 (1)	40 (2)	90 (1)s
Marital Status	Never married	517 (17)	321 (16)	838 (16)
	Currently married	2340 (76)	1535 (76)	3875 (76)
	Separated/Divorced/Widowed	216 (6)	164 (7)	380 (7)
	Unknown	23 (1)	10 (1)	33 (1)
Occupation	Government employee	99 (3)	88 (4)	187 (4)
	Non-government employee	551 (18)	406 (20)	957 (19)
	Self-employed	817 (26)	473 (23)	1290 (25)
	Unpaid	1493 (49)	995 (49)	2488 (49)
	Unemployed	131 (4)	65 (3)	196 (3)

Figures in parenthesis indicate percentages; *SC* scheduled caste, *OBC* other backward castes; SCs and STs are groups of historically disadvantaged people in India recognised by the Constitution of India; OBC is a collective term used by the Constitution of India to classify castes which are socially and educationally disadvantaged although they are considered to be at a less disadvantageous position than SCs and STs; General category is a term used in India to denote a group other than OBCs, SCs and STs and are considered socially, educationally, and economically advanced; social group and marital status are unknown because participants refused to answer these questions; Unpaid includes student, homemaker and retired

### Fruit and vegetable intake

Mean number of servings of fruits and/or vegetables per day was found to be 2.3 and 2.2 in urban and rural areas respectively. There was no urban rural difference in both sexes. Overall 95.8 % (94.6–97.0) of participants took less than five servings of fruits and/or vegetables on an average per day with no significant differences across urban-rural sub groups in both sexes Table 2.

**Table 2:** Urban rural differences in dietary practices, STEPS Survey, Punjab, India 2014-15

Variables	Males (*N* = 2381)		Females (*N* = 2746)		Total (*N* = 5127)	
	Urban	Rural	Urban	Rural	Urban	Rural
	*n* = 943	*n* = 1438	*n* = 1088	*n* = 1658	*n* = 2031	*n* = 3096
Mean number of servings of fruits and/or vegetable/day	2.3 (2.1–2.5)	2.4 (2.2–2.5)	2.2 (2.1–2.4)	2.0 (1.9–2.2)	2.3 (2.1–2.4)	2.2 (2.1–2.3)
^§^Mean number of servings of fruits/day	0.8 (0.7–1.0)	0.7 (0.6–0.7)	0.8 (0.7–1.0)	0.6 (0.5–0.7)	0.8 (0.7–1.0)	0.6 (0.5–0.7)
Ate less than 5 servings of fruits & vegetables/day	95.0 (92.5–97.4)	95.7 (94.0–97.4)	95.7 (92.8–98.6)	96.6 (95.1–98.1)	95.3 (93.1–97.5)	96.4 (95.1–97.6)
Always/often add salt before/when eating	9.5^***^ (6.0–13.1)	17.7 (13.6–21.7)	8.5^**^ (6.9–10.0)	13.3 (10.2–16.8)	9.1* (6.8–11.4)	15.6 (12.3–18.9)
Always or often eat processed foods high in salt	11.8 (7.2–16.3)	18.0 (13.8–22.2)	15.4 (9.4–21.5)	11.2 (8.2–14.0)	13.4 (8.9–17.9)	14.4 (11.7–17.1)

Estimates are weighted for age, sex, and non-response; One standard serving of fruits or vegetables is equivalent to 80 g; data in the table are presented as mean (95 % confidence interval) or % (95 % confidence interval); ^§^data for this variable were not available for 317 participants; ^*^ *p* <0.05 compared to rural subjects; ^**^ *p* < 0.05 compared to rural females; ^***^ *p* <0.05 compared to rural males;

### Dietary salt intake

About 12.8 % (10.5–15.2 %) of the population always/often add salt before/when eating (rural (15.6 %) significantly more than urban (9.1 %)). This urban-rural difference was significant in both sexes with higher salt intake among rural subjects. Table 2 Around 40 % of the population (48 % urban and 33 % rural) considered lowering the salt in the diet as very important. Significantly higher proportion of urban residents (60.9 %, 54.3–67.4 %) felt that consuming too much salt could cause serious health problems than rural residents (45.1 %, 40.4–49.8 %) (data not tabulated).

### Physical activity

Nearly 29.2 % and 32.6 % of respondents reported light levels of physical activity in urban and rural areas respectively (*p* > 0.05). Females reported significantly higher levels of light physical activity than males in both urban and rural settings. On the other hand, males reported significantly higher proportion of vigorous-intensity physical activity compared to females in both the settings Table 3.

**Table 3:** Urban rural differences in levels of physical activity, STEPS Survey, Punjab, India, 2014-15

Variables	Males (*N* = 2381)		Females (*N* = 2746)		Total (*N* = 5127)	
	Urban	Rural	Urban	Rural	Urban	Rural
	*n* = 943	*n* = 1438	*n* = 1088	*n* = 1658	*n* = 2031	*n* = 3096
Physical activity						
Light	22.6^***^ (17.2–28.0)	22.2^**^ (16.0–28.4)	37.5 (30.0–45.0)	44.1 (37.3–51.0)	29.2 (23.1–35.3)	32.6 (26.3–38.8)
Moderate	45.0 (41.3–48.7)	43.5 (39.0–48.0)	51.2^**^ (44.9–57.5)	38.7 (33.5–44.1)	47.7 (44.0–51.5)	41.3 (37.0–45.6)
Vigorous	32.4^*** ^(27.8–37.0)	34.3^**^ (28.2–40.3)	11.3 (9.0–13.7)	17.1 (12.0–22.2)	23.1 (19.5–26.6)	26.2 (21.0–31.2)
At least 150 min/week of moderate activity or equivalent (at least 600 MET–minute/week)	87.7 (83.5–91.8)	85.7^** ^(81.0–90.4)	80.8 (73.4–88.2)	70.8 (64.7–77.0)	84.7 (79.4–90.0)	78.8 (73.6–84.0)
Percentage inactive in the following domains						
Work	24.6 (16.3–32.8)	22.5 (15.4–29.5)	27.4 (16.3–38.6)	24.7 (19.2–30.2)	25.8 (17.2–34.4)	23.5 (17.8–29.2)
Transport	18.0 (13.5–22.5)	18.3^** ^(13.1–23.6)	24.1 (14.4–33.7)	32.8 (25.1–40.6)	20.6 (14.1–27.1)	25.1 (18.9–31.3)
Recreation	84.9^***^ (79.1–90.7)	87.2^**^ (84.1–90.2)	94.3 (92.0–96.7)	95.2 (93.9–96.6)	89.0 (85.2–92.8)	90.9 (89.0–92.8)
No vigorous activity	34.7^***^ (27.4–42.1)	43.5^**^ (35.5–51.6)	6.3^**^ (4.1–8.5)	19.1 (13.9–24.4)	22.3 (17.2–27.4)	32.2 (25.8–38.5)
Mean minutes spent daily towards						
Work	87.7 (83.5–91.8)	85.7^**^ (81.0–90.4)	80.8 (73.4–88.2)	70.8 (64.7–77.0)	84.7 (79.4–90.0)	78.8 (73.6–84.0)
Transport	53.0^*** ^(45.8–60.1)	50.2^**^ (43.3–57.2)	36.6 (29.4–43.7)	34.3 (27.3–41.4)	45.8 (39.5–52.1)	42.8 (36.4–49.2)
Recreation	7.4^*** ^(4.3–10.5)	8.7^**^ (6.3–11.0)	1.7 (1.1–2.4)	2.4 (1.2–3.7)	5.2 (3.3–7.2)	5.4 (4.1–6.8)
Sedentary activity	237.8 (219.7–256.0)	208 (193.1–222.8)	232.7 (212.7–252.7)	237.2 (216.0–258.4)	235.6 (218.4–252.8)	221.6 (206.1–237.2)

Estimates are weighted for age, sex, and non-response; data in the table are presented as mean (95 % confidence interval) or % (95 % confidence interval); ^*^ *p* <0.05 compared to rural subjects; ^**^ *p* < 0.05 compared to rural females; ^***^ *p* <0.05 compared to urban females

On comparing the percentage of respondent who are sufficiently active i.e reporting at least 150 min of moderate activity or equivalent of physical activity per week, which is the minimal duration recommended by the WHO, no significant difference was observed among rural and urban populations in both sexes, although rural males were more active than their female counterparts Table 3.

No significant urban rural difference was noted in both sexes in all three domains of physical activity such as work, transport and recreation. More rural females (19.1 %) were found to be not engaged in vigorous activity than the urban females (6.3 %) (*p* < 0.001). In the transport domain, rural females (32.8 %) were more inactive than rural males (18.3 %). In the recreation domain, females were more inactive than males in both the settings. On an average, 228.0 (215.9–239.2) minutes were spent in sedentary activities daily with no significant urban rural differences in both sexes. The mean time spent in transport and recreation was significantly higher among males compared to females in both rural and urban settings Table 3.

### Obesity

Overweight (BMI > 25–29.9) was observed in 28.6 % (26.3–30.9) and obesity (BMI > 30) in 12.8 % (11.2–14.4) of participants with no urban rural difference. If Asian cut offs are used (overweight (BMI: 22.9–27.49) and obesity (BMI > 27.49), the proportion of overweight and obese was higher with 33 % of adult population as overweight and 25 % as having obesity.

Overall no urban rural difference was observed in mean BMI and prevalence of overweight, obesity and abdominal obesity among males. Significantly higher prevalence of obesity was seen among urban females (34.3 %) as compared to rural females (23.2 %) (*p* < 0.001) with asian cut offs. Among males no urban rural difference was observed. The prevalence of abdominal obesity was 75 % in urban and 71 % in rural areas. Abdominal obesity was found to be significantly higher among females in both the settings compared to males (*p* < 0.001) Table 4.

**Table 4:** Urban rural differences in body mass index and abdominal obesity, STEPS Survey, Punjab, India, 2014-15

Variables	Males (*N* = 2326)		Females (*N* = 2706)		Total (*N* = 5032)	
	Urban	Rural	Urban	Rural	Urban	Rural
	*n* = 923	*n* = 1403	*n* = 1072	*n* = 1634	*n* = 1995	*n* = 3037
Mean BMI (in kg m^−2^)	24.2^***^ (23.4–25.0)	23.8 (23.4–24.2)	25.7^**^ (25.3–26.2)	24.1 (23.7–24.7)	24.9* (24.3–25.5)	23.9 (23.7–24.2)
Overweight (Asian cut off)	35.3 (31.5–39.2)	33.0 (28.5–37.3)	28.9 (26.2–31.7)	33.0 (29.1–36.6)	32.6 (30.1–35.0)	32.8 (31.0–37.0)
Obesity (Asian cut off)	23.2 (16.0–30.6)	22.1 (18.0–26.0)	34.3** (30.4–38.1)	23.2 (20.2–26.2)	28.0 (22.6–33.5)	22.6 (19.7–25.4)
Overweight (WHO cut off)	29.1 (23.4–34.9)	26.1 (22.4–29.7)	30.1 (25.7–35.1)	29.3 (25.8–32.8)	29.7 (25.4–34.0)	27.6 (24.9–30.2)
Obesity (WHO cut off)	10.6*** (5.9–15.3)	11.6 (9.2–14.1)	19.9 (17.2–22.6)	11.4 (8.9–13.9)	14.6 (11.8–17.5)	11.5 (9.8–13.3)
Abdominal Obesity	50.4^*** ^(42.8–58.0)	48.4^** ^(43.8–52.9)	72.8 (67.7–78.0)	67.0 (64.1–70.0)	64.1 (58.5–70.0)	59.1 (56.5–62.2)

Estimates are weighted for age, sex, and non-response; *BMI* body mass index; According to WHO Overweight is defined as BMI > = 25 kg/m^2^ and <30 kg/m^2^. Obesity defined as BMI > = 30 kg/m^2^; According to Asian cut off overweight is defined as BMI > = 23 kg/m^2^ and <27.5 kg/m^2^; obesity defined as BMI > =27.5 kg/m2; abdominal obesity defined waist circumference >90 cm for men and >80 cm for women; data in the table are presented as mean (95 % confidence interval) or % (95 % confidence interval); ^*^ *p* <0.05 compared to rural subjects; ^**^ *p* < 0.05 compared to rural females; ^***^ *p* <0.05 compared to urban females

Obesity was significantly higher among the older age group (45–69 years), females, general caste and among the government employee and the unpaid. Females, unpaid and the unemployed, illiterates and respondents from the general caste were least engaged in physical activity Table 5.

**Table 5:** Socio-demographic characteristics associated with obesity and lack of physical activity, STEPS Survey, Punjab, India, 2014

Characteristics	Total N	Obesity N (%)	OR (95 % CI)	*P* value	Less than WHO recommended physical activity N (%)	OR (95 % CI)	*P* value
Total	5032	672 (13)			1044 (21)		
Age group in years							
18–44	3279	370 (11)	1.0	Ref	695 (21)	1.0	Ref
45–69	1753	302 (17)	1.6 (1.4–1.9)	<0.001	349 (20)	0.9 (0.8–1.1)	0.3
Gender							
Male	2326	265 (11)	1.0	Ref	334 (14)	1.0	Ref
Female	2706	407 (15)	1.4 (1.2–1.6)	<0.001	710 (26)	2.1 (1.8–2.4)	<0.001
Social group							
SC/ST	1896	198 (10)	1.0	Ref	316 (17)	1.0	Ref
Other backward caste	683	94 (14)	1.4 (1.1–1.8)	0.02	141 (21)	1.3 (1.1–1.7)	0.006
General	2363	380 (16)	1.5 (1.3–1.8)	<0.001	587 (25)	1.6 (1.4–1.8)	<0.001
Educational status							
Illiterate	1191	130 (11)	1.0	Ref	290 (24)	1.3 (1.1–1.5)	0.005
Upto primary education	1243	185 (15)	1.4 (1.1–1.8)	0.003	234 (19)	0.9 (0.7–1.1)	0.4
Upto secondary education	748	106 (14)	1.3 (1.0–1.8)	0.03	148 (20)	1.0 (0.8–1.2)	0.8
Higher education	1850	251 (14)	1.3 (1.0–1.7)	0.03	372 (20)	1.0	Ref
Occupation							
Government employee	181	34 (18)	3.4 (2.1–5.3)	<0.001	30 (17)	1.2 (0.8–1.8)	0.4
Non-government employee	948	61 (6)	1.0	Ref	135 (14)	1.0	Ref
Self-employed	1274	163 (13)	2.1 (1.6–2.9)	<0.001	193 (15)	1.1 (0.8–1.4)	0.6
Unpaid	2320	373 (16)	2.8 (2.1–3.7)	<0.001	609 (26)	2.1 (1.7–2.6)	<0.001
Unemployed	301	41 (13)	2.3 (1.5–3.5)	<0.001	77 (26)	2.1 (1.5–2.8)	<0.001

SCs and STs are groups of historically disadvantaged people in India recognised by the Constitution of India; OBC is a collective term used by the Constitution of India to classify castes which are socially and educationally disadvantaged although they are considered to be at a less disadvantageous position than SCs and STs; General category is a term used in India to denote a group other than OBCs, SCs and STs and are considered socially, educationally, and economically advanced; social group and marital status are unknown because participants refused to answer these questions; Unpaid includes student, homemaker and retired

**Fig. 1:**
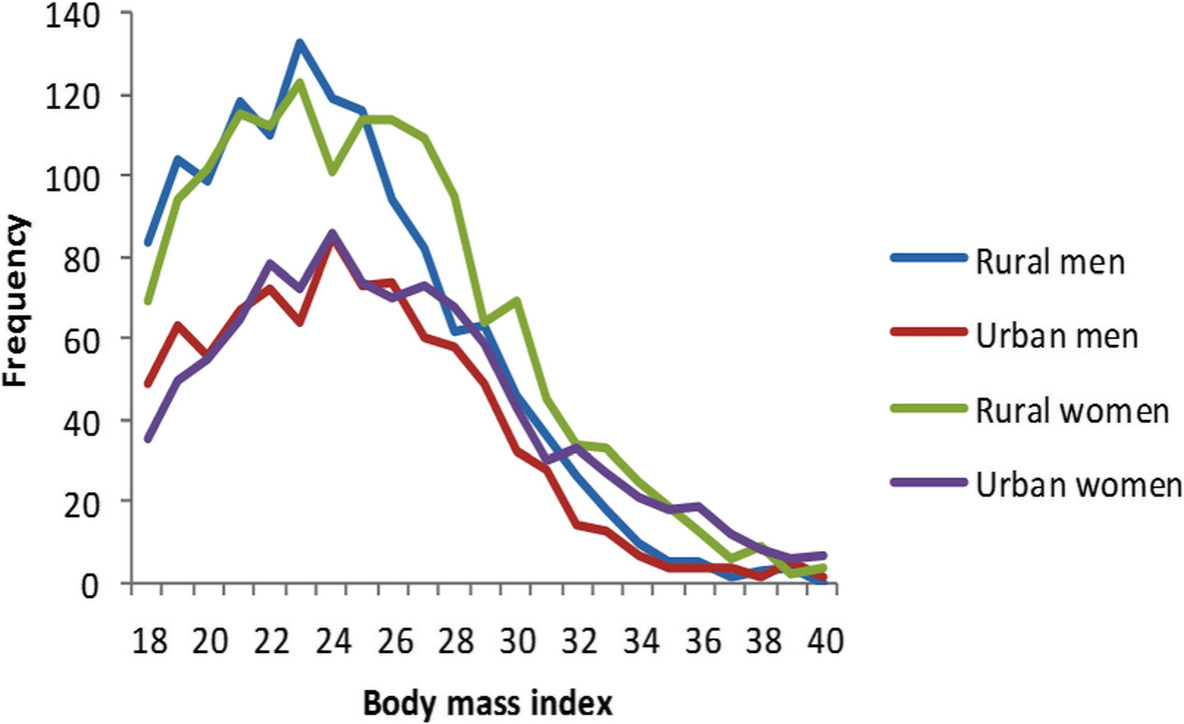
Body mass index histograms for men and women across urban and rural settings

Figure 1 clearly shows that after BMI > 25 kg/m^2^ there is minimal gap in the levels of BMI between rural and urban dwellers although there is some gender difference with females having higher BMI than males.

## Discussion

Overall a very high proportion of respondents reported inadequate intake of fruits and vegetables with no gender or urban rural differences. Males were significantly more active than females. There was no urban rural differential in physical inactivity. Urban females had higher prevalence of overweight as well as obesity. Central obesity was found to be quite higher among females in both the settings compared to males.

The study reported minimal urban rural differences in dietary habits and levels of physical activity. This is in contrast to other studies in similar settings which reported higher physical inactivity in the urban areas and poor intake of fruits and vegetables in the rural areas [12–15]. In another study in India, the rural urban disparity in inactivity was least marked in Chandigarh probably because it is a highly urbanized territory and is located adjacent to two of India’s prosperous states, Punjab and Haryana [16].

According to this study, there were no overall urban rural differences in prevalence of overweight and obesity. However urban females had significantly higher proportion of obesity than their rural counterparts. Previous studies have highlighted significant urban rural differential in prevalence of overweight and obesity with urban dwellers being more overweight and obese [12, 17]. Earlier studies were conducted more than a decade ago when urbanization was on a meteoric rise. Now after two decades of rapid globalization and urbanization, the urbanized lifestyle has moved into the rural areas. This transition of lifestyle is more evident in the study area of Punjab which is a prosperous state in North India with minimal urban rural differential in terms of socioeconomic indices.

The present study reported high prevalence of abdominal obesity especially among women which is in accordance with other large scale studies conducted in different regions of the country using similar cut offs for waist circumference [18, 19]. However, in contrast to the results of this study, earlier studies have reported significant urban-rural differences which probably points towards the equalisation of the urban rural divide [17–20]. Thus, about 60 % of the population having abdominal obesity in the current study is of considerable concern because of associated metabolic and cardiovascular consequences.

The question that arises is: Are we witnessing the great Indian equalization? A credit Suisse report by Mishra et al. emphatically said that rural India is urbanising rapidly [21]. Urbanisation in India is progressing along a path very different from the conventional migration to big cities. Rural productivity growth in India has surpassed urban growth. As villages start urbanising, their consumption habits change as well. The rural consumption basket of 2010 was surprisingly similar to the urban basket of 1994 [21]. This paradigm shift is bound to adversely affect their dietary habits and their routine activities. Thus, urbanisation of rural villages might be responsible for the reduction in the urban rural difference in dietary patterns, physical activity and obesity that existed before.

The study also reported that more than one-fifth of the respondents were physically inactive (less than the minimal duration recommended by WHO. A large community based study on NCD risk factor profiling in 2005 in India showed that overall inactivity levels were only 6.8 % [22]. However recent studies have reported much higher levels of physical inactivity in India ranging from 38 to 70 % [12, 13, 16, 23, 24]. This indicates declining physical activity levels in recent times.

According to this study, recreational physical activity levels are extremely low in India with 90 % of individuals in both urban and rural areas reporting no physical activity during leisure time. This is similar to other studies in India and Vietnam which have also reported inactivity in the recreational domain to be as high as 90 % [12, 13, 25]. The high prevalence of insufficient recreational activity observed across all sub groups could reflect limited access to and availability of facilities for recreational physical activity. The study found that there was a significant difference among males and females with respect to physical activity, with males being more active in both urban and rural settings. This is in agreement with other studies which have also reported higher levels of activity in males [12, 13, 26, 27]. In line with this, the present study also showed high prevalence of abdominal obesity among females as compared to males in both the settings which might be linked to the low physical activity levels among females.

The study revealed poor intake of fruits and vegetables across all sub groups. There is substantial evidence that low consumption of fruits and vegetables increase the risk for chronic diseases [4, 28, 29]. A multi-pronged strategic approach should be adopted for promoting healthy diet and at the same time restricting the use of unhealthy diet: communication to the population on benefits of five servings of fruits and vegetable per day, provision of information at the point of sale, food labelling and the restriction of advertising and of health claims, subsidies or direct pricing encouraging healthy eating, development of state specific dietary guidelines, aggressive taxation of snacks, strict monitoring of food safety, quality and adherence to standards. Regulations need to be put in place to control the content of salt, sugar, saturated fats and trans fats in dietary products.

In most countries, dietary salt intake is well above the recommended levels [30, 31] including India [32, 33]. The majority of salt in Western diets comes from processed foods [31, 34] while in developing countries it comes from the salt that is added to food during food preparation [31]. A review of literature showed that about 20 % of the subjects usually or always used discretionary salt which is higher than the figures reported in this study [35]. The review also reported that in most studies the participants were well aware of the adverse health consequences of salt intake similar to the present study [35]. Still the dietary salt intake remains high. This is probably because of the lack of awareness of personal daily salt intake which requires consumer education programs [35].

The advantage of the study is that it employed a multi-stage stratified sampling approach using a 2011 census frame and involved the whole state of Punjab which lends generalisability to the study findings. The study followed the standard robust methodology of STEPS survey which also provides internal validity to the results of the survey.

The study has few limitations. Firstly, this being a self-reported survey, participants tend to report in socially desirable ways. For example, the less active may want to over-report activity to appear healthier. Secondly, data collection from subjects depends mainly on their recall ability which has inherent biases. Thirdly, although GPAQ used as a part of the STEPS has been found to be a reliable and valid tool, there are some issues related to its validity [36]. It has been reported that the GPAQ probably underestimates regular physical activity at work especially of housework and leisure time physical activity [12]. This might explain lower vigorous activity levels among women. Vigorous intensity physical activity might be easier to recall and therefore more accurately reported than lower intensity activity as reported in previous studies [37, 38]. The participants in this study may have had a difficult time estimating moderate intensity PA, which is known to be difficult both perceptually and cognitively [39, 40]. Another major limitation of GPAQ for assessment in India is that it does not capture the diversity of activities across cultures and by sex [41].

## Conclusion

Overall there were no significant urban/rural differences in dietary habits, physical activity and obesity. However, poor dietary practice, low physical activity reported by almost one third of participants as well as high prevalence of obesity point out the need for more aggressive promotion of information, education and communication strategies around healthy lifestyle and healthy balanced diet. The study results also highlight the urgent need to promote overall physical activity, especially recreational activities during leisure time or at workplaces by creating adequate recreational infrastructure and enabling environments. The significant impact on women as compared to men warrants gender sensitive strategies.

## Abbreviations

ANOVA: Analysis of variance
BMI: Body mas index
CEB: Census enumeration block
GPAQ: Global physical activity questionnaire
HDL: High density lipoproteins
LDL: Low density lipoproteins
NCD: Non communicable disease
SDGs: Sustainable development goals
